# The role of sphingosine-1-phosphate in the gut mucosal microenvironment and inflammatory bowel diseases

**DOI:** 10.3389/fphys.2023.1235656

**Published:** 2023-07-25

**Authors:** Fei Zou, Su Wang, Mengmeng Xu, Zengrong Wu, Feihong Deng

**Affiliations:** ^1^ Department of Gastroenterology, The Second Xiangya Hospital, Central South University, Changsha, Hunan, China; ^2^ Research Center of Digestive Disease, Central South University, Changsha, Hunan, China

**Keywords:** sphingosine-1-phosphate, S1P-S1PR signaling pathway, intestinal epithelial barrier, immune dysfunction, inflammatory bowel disease

## Abstract

Sphingosine-1-phosphate (S1P), a type of bioactive sphingolipid, can regulate various cellular functions of distinct cell types in the human body. S1P is generated intracellularly by the catalysis of sphingosine kinase 1/2 (SphK1/2). S1P is transferred to the extracellular environment via the S1P transporter, binds to cellular S1P receptors (S1PRs) and subsequently activates S1P-S1PR downstream signaling. Dysbiosis of the intestinal microbiota, immune dysregulation and damage to epithelial barriers are associated with inflammatory bowel disease (IBD). Generally, S1P mainly exerts a proinflammatory effect by binding to S1PR1 on lymphocytes to facilitate lymphocyte migration to inflamed tissues, and increased S1P was found in the intestinal mucosa of IBD patients. Notably, there is an interaction between the distribution of gut bacteria and SphK-S1P signaling in the intestinal epithelium. S1P-S1PR signaling can also regulate the functions of intestinal epithelial cells (IECs) in mucosa, including cell proliferation and apoptosis. Additionally, increased S1P in immune cells of the lamina propria aggravates the inflammatory response by increasing the production of proinflammatory cytokines. Several novel drugs targeted at S1PRs have recently been used for IBD treatment. This review provides an overview of the S1P-S1PR signaling pathway and, in particular, summarizes the various roles of S1P in the gut mucosal microenvironment to deeply explore the function of S1P-S1PR signaling during intestinal inflammation and, more importantly, to identify potential therapeutic targets for IBD in the SphK-S1P-S1PR axis.

## 1 Introduction

Inflammatory bowel disease (IBD) is defined by gastrointestinal nonspecific chronic inflammation and includes Crohn’s disease (CD) and ulcerative colitis (UC). IBD occurs frequently in Western countries, with a rising incidence in newly industrialized countries in Africa, Asia and South America; furthermore, it has become a global health issue that places increasing financial and medical burdens on people (Ng et al., 2017). The pathogenesis of IBD is unclear, and it is associated with genetic susceptibility, environmental changes, intestinal microbiota imbalance and immune dysregulation (Xavier and Podolsky, 2007). Reducing mucosal inflammation and promoting intestinal mucosal wound healing are considered the main goals of IBD treatment. Conventional medicines such as 5-aminosalicylates, glucocorticoids and immunosuppressants do not have satisfactory anti-inflammatory effects and have some side effects. Currently, clinically applied biologic agents, including anti-TNF-α agents, anti-integrin agents and anti-interleukin 12/23 agents, have obtained tremendous advances, but there are still several deficiencies, such as individual differences in efficacy, failures in response and high recurrence rates (Al-Bawardy et al., 2021). Therefore, novel therapeutic agents targeting different pathways are urgently needed for IBD therapy. As representatives, S1P receptor modulators have received much attention.

Abnormal lipid metabolism is involved in the pathogenesis of IBD (Alhouayek et al., 2021). Metabolomic analysis showed that sphingomyelin (SM) levels in the ileums of TNF^△ARE/WT^ mice that spontaneously experienced CD-like symptoms were significantly higher than those in WT control mice, indicating that SM may be associated with IBD pathogenesis (Baur et al., 2011). Sphingosine-1-phosphate (S1P) is a type of bioactive sphingolipid. The synthesis of S1P is catalyzed by sphingosine kinase 1/2 (SphK1/2) from sphingosine (Sph) intracellularly and is transferred to the extracellular environment via the S1P transporter (Spiegel et al., 2019). Secreted S1P can bind to five S1P receptors (S1PRs) on the cell membrane of various cell types and further activate downstream signaling, such as Rho/ROCK, PI3K/Akt and ERK, which regulate cell survival, proliferation, migration and apoptosis (Baeyens and Schwab, 2020; Gomez-Larrauri et al., 2020). Plasma S1P levels in UC patients are higher than those in healthy controls (Sun et al., 2019a). The SphK1 inhibitor PF-543 had an inhibitory effect in a DSS-induced colitis mouse model, indicating that the SphK-S1P axis is closely associated with the progression of IBD (Liu and Jiang, 2020).

The most studied mechanism of action of S1P is that it affects the inflammatory immune response by binding to S1PR1 on lymphocytes and regulating the migration of T and B cells out of the peripheral lymphoid tissue (Matloubian et al., 2004). S1PR modulators, which inhibit S1P-S1PR1 signaling, treat IBD by inhibiting the infiltration of lymphocytes into the inflamed intestinal lamina propria (Deguchi et al., 2006; Pérez-Jeldres et al., 2019). S1PR modulators have recently become a hotspot in IBD therapy, and they can induce and help maintain remission in moderate to severe UC and CD with marked improvement of clinical symptoms and few adverse events (Wang et al., 2022a). Beyond the role of S1P in lymphocyte migration, studies have also reported that S1P can be related to changes in the intestinal bacterial composition in IBD (Sun et al., 2019a), and it could affect mucosal inflammation by regulating the intestinal epithelial barrier (Dong et al., 2015) and the phenotypes of immunocytes such as macrophages (Wang et al., 2022b). Thus, in addition to regulating lymphocyte egress, the S1P-S1PR pathway is also related to the gut microbiota, intestinal epithelial cells (IECs) and immune cells in the lamina propria. It may be possible to develop additional drugs that target the S1P-S1PR pathway for intestinal disease. This review discusses the relationship between S1P and the gut microbiota, its various functions in different cell types in the intestinal mucosa, including epithelial cells and innate immune cells, and its roles in IBD pathogenesis. We also investigated the effects of the S1P-S1PR pathway in IBD therapeutics in hopes of developing novel drugs for IBD.

## 2 S1P: metabolism, receptors and functions

**FIGURE 1:**
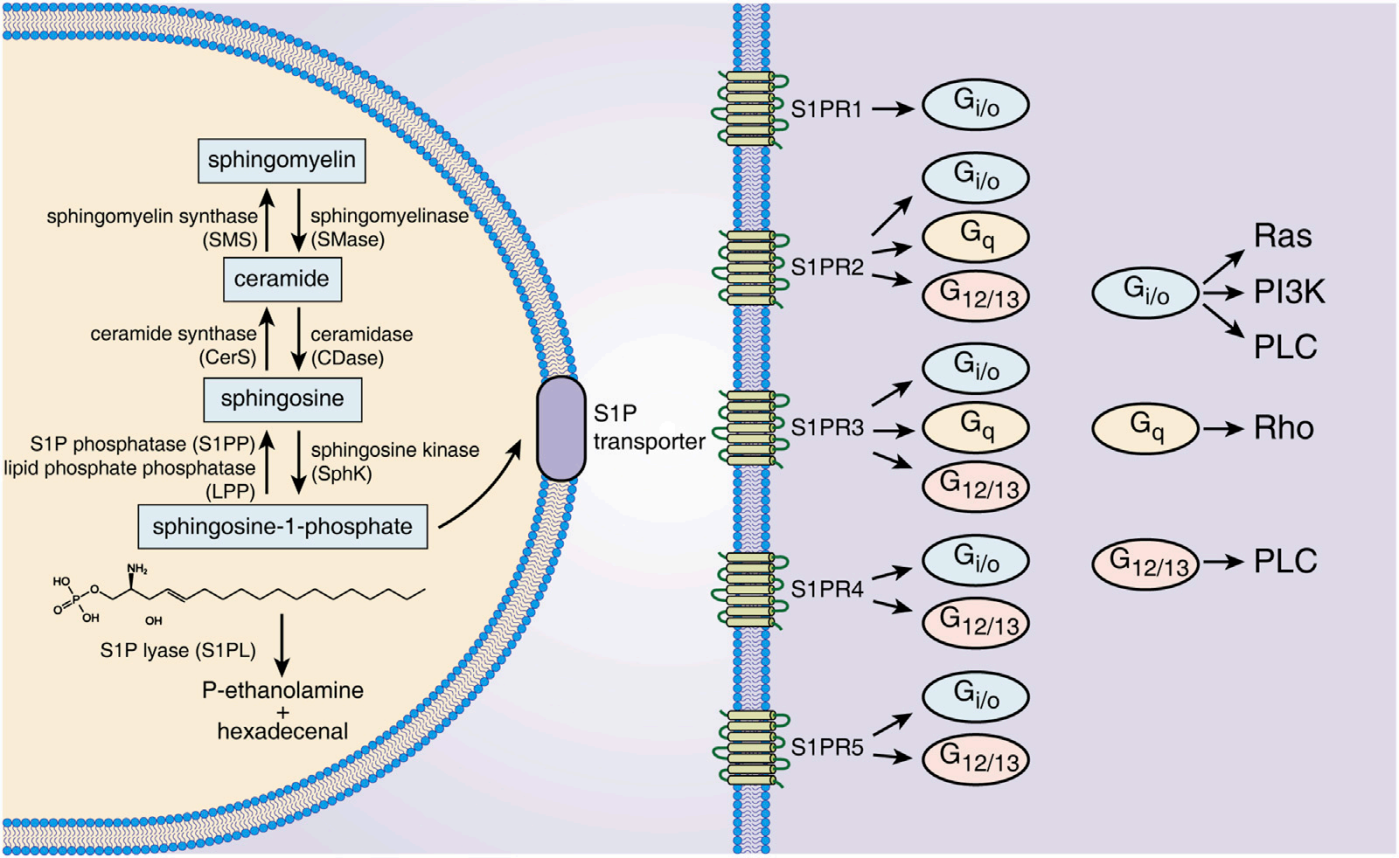
S1P metabolism, receptors and downstream signals. S1P is generated from Sph by intracellular SphK1/2 catalysis and is dephosphorylated to Sph by S1PP or irreversibly degraded by S1PL. S1P is transported out of cells through specific transporters on the cell membrane and binds to five S1PRs of the same or other cells. Different S1PRs couple to different G proteins and activate specific downstream signals. Abbreviations: S1P, sphingosine-1-phosphate; SMS, sphingomyelin synthase; SMase, sphingomyelinase; CerS, ceramide synthase; CDase, ceramidase; S1PP, sphingosine-1-phosphate phosphatase; LPP, lipid phosphate phosphatase; SphK, sphingosine kinase; S1PL, sphingosine-1-phosphate lyase; S1PR1∼5, sphingosine-1-phosphate receptor 1–5; Ras, rat sarcoma protein; PI3K, phosphatidylinositol 3-kinase; PLC, phospholipase C; Rho, ras homolog.

Sphingolipids are a class of bioactive lipids containing a backbone of sphingoid bases, consisting of SM, ceramide (Cer), Sph, S1P, etc. (Gomez-Larrauri et al., 2020). Sphingomyelinase catalyzes SM to generate Cer in the cell membrane or lysosome. The latter is then hydrolyzed by ceramidase to form Sph (Fyrst and Saba, 2010). S1P is generated intracellularly by phosphorylation of Sph by SphK. SphK has two isoenzymes called SphK1 and SphK2 (Pyne et al., 2009). Although SphK1/2 are highly homologous, they are different in their subcellular locations and functions. For example, SphK1 is mainly in the cytosol, while SphK2 exists in intracellular compartments and the cell nucleus (Maceyka et al., 2012). Overexpression of SphK1 promotes cell growth and reduces cell apoptosis, while SphK2 inversely promotes apoptosis (Spiegel and Milstien, 2003). Several studies have indicated that SphK1 levels are positively correlated with the production of secreted S1P (González-Fernández et al., 2017). SphK2 can inhibit SphK1 production, and SphK2 knockout significantly increases S1P expression in serum and colon tissue. S1P has three different modes of degradation that act either intracellularly or extracellularly. S1P phosphatase 1/2 (S1PP1/2) and S1P lyase (S1PL) catalyze S1P degradation intracellularly (Fyrst and Saba, 2010), and lipid phosphate phosphatase dephosphorylates S1P in the extracellular environment (Tang and Brindley, 2020). The activities of SphK1/2, S1PP and S1PL together determine the level of intracellular and secreted S1P, making these proteins potential therapeutic targets for S1P-associated diseases (Figure 1).

S1P regulates cell functions by binding with S1PRs and then activating downstream signals. Intracellularly generated S1P binds to S1PRs on the membrane of targeted cells in autocrine, paracrine and/or endocrine ways, which is called “inside-out signaling” of S1P (Deng et al., 2015). Because S1P has a polar head group, it cannot freely pass through the cell membrane and requires specific transporters on the cell membrane (Nagahashi et al., 2014). The following two types of S1P transporters have been identified: ATP-binding cassette transporters and spinster 2 (Spns2) transporters. Inhibition of these transporters can effectively decrease S1P secretion, and these transporters have been considered a potential target for several immune disease therapies, such as that for IBD, multiple sclerosis and rheumatoid arthritis (Donoviel et al., 2015). The five receptors of S1P (S1PR1-5) are all G protein-coupled receptors located on the cell membrane. S1PR1/2/3 are widely expressed, while S1PR4/5 are mainly expressed in lymph tissue, hemopoietic tissue and the central nervous system (Danese et al., 2018). Different S1PRs couple to different G proteins as follows: S1PR1 couples to Gi/o, S1PR2 and S1PR3 can both couple to G12/13, Gi/o and Gq, and S1PR4 and S1PR5 both couple to Gi/o and G12/13 (Spiegel and Milstien, 2003). By the interaction of S1PRs/G proteins, distinct downstream signals, including that of the ERK, Ras, Rho and PI3K pathways, are activated (Spiegel and Milstien, 2003). The S1P-S1PR1 pathway mainly regulates cell growth, proliferation, migration and differentiation, as well as affecting angiogenesis and lymphocyte egression, which is in opposition to the roles of the S1P-S1PR2 pathway (Wang et al., 2022a). S1P-S1PR3 signaling is closely related to immunocyte chemotaxis, recruitment and maturation (Li et al., 2021). S1PR4 could influence vasoconstriction and immunocyte differentiation, and S1PR5 affects natural killer cell migration (Nielsen et al., 2017). Thus, the distinct biological roles of S1PRs in tissues indicate the wide and complicated effects of S1P-S1PR signaling in different target cells.

## 3 S1P in IBD pathogenesis

The intestinal mucosal microenvironment mainly consists of the gut microbiota, the epithelial barrier, and innate and adaptive immune cells (Öhman et al., 2015). Bacteria-cell crosstalk and cell‒cell crosstalk in the epithelium are of great importance to intestinal homeostasis, and disordered crosstalk contributes to an abnormal microenvironment that is manifested by intestinal dysbacteriosis, increased intestinal permeability and immune dysfunction, all of which are acknowledged hallmarks of IBD pathogenesis (Zaidi et al., 2020). Due to the widespread expression of S1PRs in different cell types and the increased S1P expression in IBD (Karuppuchamy et al., 2017), in the gut mucosal microenvironment, S1P may have important regulatory roles in gut microbial and cellular function, thereby participating in the development and progression of IBD.

### 3.1 Expression of S1P in intestinal tissue

S1P expression is different between normal and inflammatory intestines. In normal intestinal tissue, the level of S1P is low due to lower expression of SphK1 and higher expression of S1PL (Suh and Saba, 2015; Karuppuchamy et al., 2017). Of note, SphK1 is overexpressed in the intestinal mucosa of UC and CD patients (Karuppuchamy et al., 2017). The levels of S1P either in the epithelium or lamina propria of the colon are increased in TNBS-induced mouse models (Maines et al., 2010). The expression of S1PRs is different in various cell types in the intestine (Table 1). Under homeostasis, all S1PRs except S1PR4 are present in IECs; among them, S1PR2 has the highest expression, followed by S1PR3 (Chen et al., 2017). Bone marrow-derived macrophages express mainly S1PR1 and S1PR2 with little expression of S1PR3/4 and no expression of S1PR5 (Michaud et al., 2010; Skoura et al., 2011). Immature dendritic cells (DCs) express all five S1PRs, and S1PR1/2/3 are comparatively highly expressed in immature DCs (Xiong et al., 2019). Natural killer cells (NK cells) express all S1PRs except S1PR3 (Drouillard et al., 2018), and neutrophils express all five S1PRs with relatively high expression of S1PR1/4 (Allende et al., 2011). Upon inflammation, epithelial cell damage as well as phenotypic changes in immune cells occur, such as neutrophil activation and transformation of macrophages and DCs into a proinflammatory phenotype, and the expression of S1PRs in various cell types is also changed during this period. A significant increase in S1PR2 expression was observed in macrophages in the intestinal mucosa of UC patients and mouse models (Wang et al., 2022b). LPS stimulates S1PR1/2/3 overexpression in DCs and neutrophils (Perez et al., 2019; Xiong et al., 2019).

**TABLE 1:** S1PR expression in intestinal cells under homeostasis or inflammation.

	Expression of S1PRs under homeostasis					Expression of S1PRs under inflammation				
	IECs	Mφ	DCs	NK cell	N	IECs	Mφ	DCs	NK cell	N
S1PR1	+	+++	+++	+	+++	-	-	↑	-	↑
S1PR2	+++	+++	+++	+	++	-	↑	↑	-	↑
S1PR3	++	+	+++	-	++	-	-	↑	-	↑
S1PR4	-	+	++	++	+++	-	-	-	-	-
S1PR5	++	-	++	+	+	-	-	-	↑	-

Note: The profile of S1PR expression varies in different types of cells in the intestine.

“↑” represents the increased expression of specific S1PR in specific cells under inflammation.

“-” represents no level change or unknown condition.

Abbreviations: IECs, intestinal epithelial cells; Mφ, macrophages; DCs, dendritic cells; N, neutrophils; S1PR1∼5, sphingosine-1-phosphate receptor 1–5.

The expression of S1PP and S1PL is also different in normal and inflammatory intestines. S1PL is highly expressed in intestinal tissues under homeostasis and is decreased in colitis model mice (Degagné et al., 2014; Karuppuchamy et al., 2017). Karuppuchamy et al. demonstrated that S1PP2 mRNA expression was downregulated in the intestinal tissues of UC patients and in a DSS-induced colitis mouse model. The expression of S1PP1 mRNA was also downregulated in a DSS-induced colitis mouse model (Karuppuchamy et al., 2017). In addition, the expression of the S1P transporter Spns2 is increased in the intestinal mucosa of UC and CD patients, which may contribute to the high S1P levels in the inflamed intestine (Karuppuchamy et al., 2017).

### 3.2 S1Ps interaction with gut microbiota

Microbiota dysbiosis is an important pathogenesis of IBD that can cause chronic intestinal inflammation. The therapeutic effects of antibiotics, probiotics and fecal microbiota transplantation on IBD reflect the value of therapeutic strategies targeting the gut microbiota (Sartor, 2004; Narula et al., 2017). *Firmicutes* and *Bacteroidota* dominate the intestinal flora of healthy adults (Lloyd-Price et al., 2019). The microbiota dysbiosis of IBD patients is characterized by decreased commensal bacterial diversity, increased abundance of *Proteobacteria* and decreased abundance of *Firmicutes* and *Bacteroidota* (Lloyd-Price et al., 2019). The abundances of *Prevotellaceae* and *Ruminococcaceae* were decreased in the gut of DSS-induced colitis model mice with an increased abundance of *Desulfovibrio* (Xie et al., 2019; Liu et al., 2020).

At present, there are limited studies on the relationship between the S1P-S1PR pathway and gut microbiota. Miao et al. reported that SphK2 (a major risk factor for IBD) knockout mice showed an increased abundance of *Firmicutes* and *Verrucomicrobia* through 16S rDNA amplicon sequencing of cecal contents (Miao et al., 2022). Uptake of bacteria-secreted particles (BSPs) secreted from enterotoxigenic *Bacteroides fragilis* by IECs increased SphK1/2 levels in IECs and elevated S1P levels in IEC-derived exosomes. Meanwhile, incubation of IECs with the supernatant of macrophages ingesting enteropathogenic BSPs also increased the expression of SphK1/2 in IECs (Deng et al., 2015). The effects of BSPs secreted from enterotoxigenic *B. fragilis* may be associated with the high content of sphingolipids in the cell membrane of *B. fragilis* (Sears, 2009; An et al., 2011). Moreover, the level of plasma S1P in UC patients was negatively correlated with the abundance of beneficial *Roseburia* in feces but was positively correlated with the abundance of pathogenic *Klebsiella* and *Escherichia-Shigella* (Sun et al., 2019a). In conclusion, upregulated SphK-S1P expression can aggravate gut inflammation by modulating the composition of the gut microbiota, resulting in the development of IBD, and intestinal bacteria can also affect the level of S1P in IECs through mutual interactions.

### 3.3 S1Ps interaction with the intestinal epithelial barrier

The intestinal epithelial barrier is the most important component of the gut mucosal barrier and is the key to defense against the invasion of various pathogens into the intestinal mucosa (Odenwald and Turner, 2017). Epithelial cells, which consist of absorptive cells, goblet cells, neuroendocrine cells, Paneth cells and intestinal stem cells (ISCs), are the main constituents of the intestinal epithelial barrier and play indispensable roles in the maintenance of intestinal homeostasis (Kurokawa et al., 2020). Damage to the intestinal epithelial barrier in IBD results in the translocation of bacteria into the lamina propria of the mucosa, thus initiating a mucosal immune response, causing aggravated intestinal inflammation, further promoting the death of IECs and barrier dysfunction (Wang et al., 2022a). Achieving mucosal healing is an important strategy for the treatment of UC (Kucharzik et al., 2020).

#### 3.3.1 S1P-S1PR signaling helps maintain the intestinal epithelial barrier by strengthening intercellular junctions

Intercellular junctions are the keystone of the intestinal epithelial barrier. Tight junctions (including occludin, claudin protein family and ZO protein family) and adherens junctions (including cadherin and α/β/γ-catenin) are two important components of intercellular junctions. Intestinal epithelial barrier damage caused by intercellular junction dysfunction is an essential factor in IBD pathogenesis (Kaminsky et al., 2021). Upon homeostasis, S1P binding to S1PR2 on the surface of IECs can induce increased expression of intercellular junction-associated proteins such as E-cadherin and ZO-1 and promote E-cadherin localization to the edge of cells, thus strengthening the intestinal epithelial barrier and reducing the permeability of the intestinal epithelium (Greenspon et al., 2011; Chen et al., 2017). Chen et al. found that S1PR2 siRNA could damage intercellular tight junctions by electron microscopy and showed that S1PR2 siRNA significantly increased the fluorescein permeability of the Caco-2 cell layer. The protective effect of S1PR2 on the intestinal epithelium was correlated with activation of ERK1/2 and SphK2 and decreased expression of HDAC1/2 (Chen et al., 2022). Compared with DSS-treated WT mice, S1PR2 knockout mice presented with increased intestinal permeability and elevated levels of IFN-γ, IL-6 and TNF-α in colonic tissues (Chen et al., 2018). The S1PR1 agonist SEW2871 can restore the expression of occludin and ZO-1, which are destroyed by inflammation (Dong et al., 2015). In addition, S1PP2 knockout mice had increased resistance in a DSS-induced colitis mouse model. Compared with WT mice, S1PP2^−/−^ mice had higher intestinal epithelial barrier integrity (manifested as increased expression of E-cadherin) and lower levels of TNF, IL-1β and IL-6 in intestinal tissue in the DSS-induced colitis mouse model (Huang et al., 2016). These results indicate that the S1P-S1PR pathway protects the epithelial barrier by strengthening the cell junctions between IECs. Strengthening of the epithelial barrier reduces the invasion of pathogens into the mucosa, thus inhibiting the activation of immune cells in the lamina propria and reducing mucosal inflammation in IBD.

#### 3.3.2 S1P-S1PR signaling inhibits IEC apoptosis and promotes IEC proliferation

Physiological apoptosis of IECs usually occurs at the top of villi, while apoptosis at crypts is significantly elevated in IBD (Patankar and Becker, 2020). The S1P-S1PR pathway is characterized as antiapoptotic and prosurvival in epithelial cells. The number of TUNEL-positive IECs in the colonic mucosa of S1PP2^−/−^ mice after DSS treatment was significantly reduced compared with that in WT mice, indicating that S1P can suppress IEC apoptosis during colitis (Huang et al., 2016). S1P-S1PR1 signaling can reduce caspase-3 activity by activating the Akt pathway and thus inhibiting IEC apoptosis induced by TNF-α/cycloheximide (Greenspon et al., 2009). It was also confirmed that an S1PR1 agonist can inhibit apoptosis of epithelial cells and improve epithelial barrier function to reduce colitis in an IL-10-knockout colitis mouse model (Dong et al., 2015). Thus, the S1P-S1PR pathway can protect the intestinal epithelial barrier by suppressing IEC apoptosis during gut inflammation.

IECs have a strong ability to regenerate after damage. Inflammatory factors secreted by inflammatory cells can bind to receptors on IECs and activate ISC-associated pathways such as the Hippo or Wnt/β-catenin pathway, which promotes ISC proliferation, migration and differentiation and further accelerates regeneration of the intestinal epithelium (Snippert et al., 2010; Giakountis et al., 2016; Deng et al., 2018). Previous studies have indicated that the S1P-S1PR pathway can promote the proliferation of multiple types of progenitor/stem cells, including endothelial progenitor cells and neural progenitor/stem cells (Ng et al., 2018). S1P can promote IEC proliferation and migration under gut homeostasis. Increased SphK1 expression promotes c-Myc translation by promoting checkpoint kinase 2 expression and HuR phosphorylation, ultimately leading to accelerated IEC proliferation (Jiang et al., 2013; Chen et al., 2017). S1P can also activate p38MAPK to promote the proliferation of Caco-2 cells (Thamilselvan et al., 2002). For epithelial injury, colonoscopic-guided pinch biopsies in the colons of mice caused remarkable increases in SphK1 and S1P in the mucosal wound bed (Montrose et al., 2018), indicating that S1P may participate in repair of the intestinal epithelium after injury. Further studies can deeply explore the mechanism underlying this interesting finding.

### 3.4 S1Ps interaction with the innate immune system of the gut

The content of S1P is high in blood and lymphatic fluid, while it is low in cell and interstitial fluid. This significant concentration gradient of S1P between efferent lymphatic vessels and lymph nodes drives lymphocytes to transfer into the blood from peripheral lymphoid organs through S1PR1 (Hla et al., 2008). S1PR modulators can block this concentration-dependent regulation of lymphocyte migration from peripheral lymphatic organs and exert a powerful immunosuppressive effect. The effect of S1PR modulators such as ozanimod and etrasimod on IBD has been reviewed in previous studies (Hirten and Sands, 2021; Verstockt et al., 2022). Here, we will review the role of the S1P-S1PR signaling pathway in regulating innate immune cells of the gut, including neutrophils, macrophages, DCs and NK cells, beyond its regulation in adaptive immunity.

#### 3.4.1 S1P and neutrophils

Neutrophils are important components of innate immunity and are generally considered the first responders to inflammation, which is associated with the occurrence of IBD. The effects of S1P on neutrophils under IBD mainly focus on the regulation of cell migration from the bone marrow to peripheral blood and survival. Previous studies have shown that elevated SphK1-S1P in a DSS-induced colitis mouse model led to increased neutrophilia in circulation and increased neutrophil infiltration in the colon (Snider et al., 2009; Pulkoski-Gross et al., 2017). Zhao et al. showed that S1PR1/2/3 was detected in mouse bone marrow neutrophils and differentiated human neutrophil-like cells (dHL60), and S1P powerfully boosted the migration and cytoskeletal remodeling of bone marrow neutrophils through S1PR1 or S1PR2 in liver injury (Zhao et al., 2021), indicating that S1P-S1PR signaling mediated effects on neutrophil recruitment, thus triggering tissue inflammation.

The lifespan of mature neutrophils is short under physiological conditions, while that of activated neutrophils under inflammation is significantly extended, which contributes to the initiation of inflammation (Zhao et al., 2020). S1P can inhibit neutrophil apoptosis by activating Gi/o and downstream p38 MAPK signaling (Chihab et al., 2003; Lin et al., 2011). Activation of the S1P-S1PR2-Gi/o-p38 MAPK pathway can promote the formation of neutrophil extracellular traps (Zhao et al., 2020), which could aggravate and prolong intestinal inflammation and is closely related to the development of IBD (Drury et al., 2021). Moreover, S1PR1/2/3 inhibitors increase caspase-3-dependent neutrophil apoptosis (Perez et al., 2019). Therefore, S1P may promote the development of IBD by inducing neutrophil recruitment and inhibiting neutrophil apoptosis to enhance its proinflammatory activity.

#### 3.4.2 S1P and macrophages

Macrophages are important constituents of the innate immune system that can polarize into the following two phenotypes after different stimuli in the microenvironment: M1 (classically activated macrophages) and M2 (alternatively activated macrophages) (Funes et al., 2018). M1 macrophages mainly produce proinflammatory cytokines (TNF-α, IL-6, IL-23, etc.), which are involved in inflammation aggravation and tissue injury (You et al., 2016). In contrast, M2 macrophages are considered to have anti-inflammatory effects by releasing several anti-inflammatory cytokines (You et al., 2016). Dysregulation of the M1/M2 macrophage balance might be a crucial pathogenesis of IBD. Administering M2 macrophages by intravenous injection significantly alleviates colitis and accelerates wound healing (Liang et al., 2022). The S1P-S1PR pathway is also involved in the regulation of intestinal macrophage polarization. Yang et al. reported that S1PR2/3 promoted the M1 polarization of bone marrow-derived macrophages by activating the G(α)i/o/PI3K/JNK pathway (Yang et al., 2018). Wang et al. demonstrated that S1P acting on S1PR2 induced M1 polarization of intestinal macrophages through the RhoA/ROCK1 pathway in a DSS-induced colitis mouse model (Wang et al., 2022b), revealing that S1P-S1PRs contribute to aggravated intestinal mucosal inflammation by promoting the M1 polarization of macrophages and the production of proinflammatory cytokines.

The infiltration of macrophages in the intestinal mucosa of IBD patients was significantly increased (Kühl et al., 2015; Wang et al., 2022b). Newly infiltrated macrophages have high NF-κB signal activity and mainly secrete proinflammatory cytokines, leading to increased intestinal inflammation (Kühl et al., 2015). Chemokines such as CCL2 and CCL7 are involved in the recruitment of macrophages in the intestinal mucosa in IBD (Kühl et al., 2015; He et al., 2019). S1P-S1PRs can promote the migration of macrophages to local inflamed sites. Studies in the liver (Liao et al., 2018) and lung (Nagahashi et al., 2018) have shown that the S1P-S1PR1 pathway can mediate chemotactic infiltration of macrophages to S1P-rich sites. S1PR2 knockout significantly decreased the number of macrophages in atherosclerotic plaques (Skoura et al., 2011). These studies suggest that S1P-S1PRs mediate macrophage chemotaxis and migration to resident inflamed sites. For the intestinal tract, triptolide downregulated SphK1 and S1PR1/2 expression and inhibited macrophage recruitment in the colons of DSS/AOM-induced colitis-associated cancer model mice in a dose-dependent manner (Li et al., 2020). FTY720, a functional inhibitor of S1PR1, can significantly decrease the number of macrophages in the colonic mucosa of mice infected with *Citrobacter rodentium*, suggesting that S1P-S1PR1 signaling is involved in the recruitment of macrophages to the injured gut (Murphy et al., 2012). However, it should be noted that S1P-S1PR signaling is also required for macrophage emigration from inflammatory sites during inflammation regression, and inhibition of the S1P-S1PR pathway may block the restoration of tissue homeostasis (Weichand et al., 2013). Therefore, S1P-S1PRs play important roles in macrophage recruitment and emigration at different stages of inflammation, and it is necessary to pay attention to the appropriate timing of administration of drugs targeting the S1P-S1PR pathway.

#### 3.4.3 S1P and dendritic cells

DCs are specialized antigen-presenting cells that act as sentinels in the immune system. DCs mainly include conventional DCs and plasmacytoid DCs. In active IBD patients, chemokines induce the migration of plasmacytoid DCs in circulation to inflamed sites of the gut and promote the production of proinflammatory cytokines such as IL-6 and TNF-α, which play an important role in the initiation of acute inflammation in the intestine (Arimura et al., 2017; Liu et al., 2019). Many factors, such as pathogenic microbial composition, can induce the maturation and activation of DCs and stimulate the expression of costimulatory molecules such as CD86, CD80 and CD40 (Ueno et al., 2010; Mbongue et al., 2017). These costimulatory molecules bind to CD28 or CD40L on the Th0 cell membrane, which triggers DC secretion of IL-12, resulting in T-cell differentiation and activation (Mbongue et al., 2017; Bernardo et al., 2018). DC-LMP1/CD40 chimeric mice spontaneously experienced IBD-like symptoms, manifested with massive granulocyte and lymphocyte infiltration in the intestinal mucosa (Kusters et al., 2017), indicating the pathological role of DCs in IBD.

SphK1-S1P was found to be required for plasmacytoid DC activation and the production of type I IFN and proinflammatory cytokines stimulated by the Toll-like receptor 7/9 ligand (Seo et al., 2012; Mohammed et al., 2020). SphK1-S1P can stimulate the expression of MHC II and CD40/CD80/CD86 in DCs to subsequently induce T-cell activation (Jung et al., 2007; Xu et al., 2013). FTY720, a functional inhibitor of S1PR1, significantly downregulates the number of CD11c^hi^MHC II+CD4+DCs in the bone marrow of CLP mice (a human multibacterial bacteremia model) (Smirnov et al., 2017). Moreover, knockout of S1PR2 or use of its inhibitor suppressed LPS-induced DC maturation, accompanied by decreased expression of MHC II and CD86, CD80, and CD40 in DCs (Park and Im, 2019). Additionally, S1P-S1PR4 signaling is required for plasmacytoid DC progenitor cells to differentiate into plasmacytoid DCs (Dillmann et al., 2015). S1P could promote the secretion of proinflammatory cytokines, including IL-6 and IL-8, in mature DCs in the presence of stimulus (Oz-Arslan et al., 2006). These results indicate that the S1P-S1PR pathway could regulate DC maturation and activation, which are important for triggering the inflammatory response in mucosal immunity.

### 3.5 Contradiction of the role of S1P in the epithelium and lamina propria and crosstalk

**FIGURE 2:**
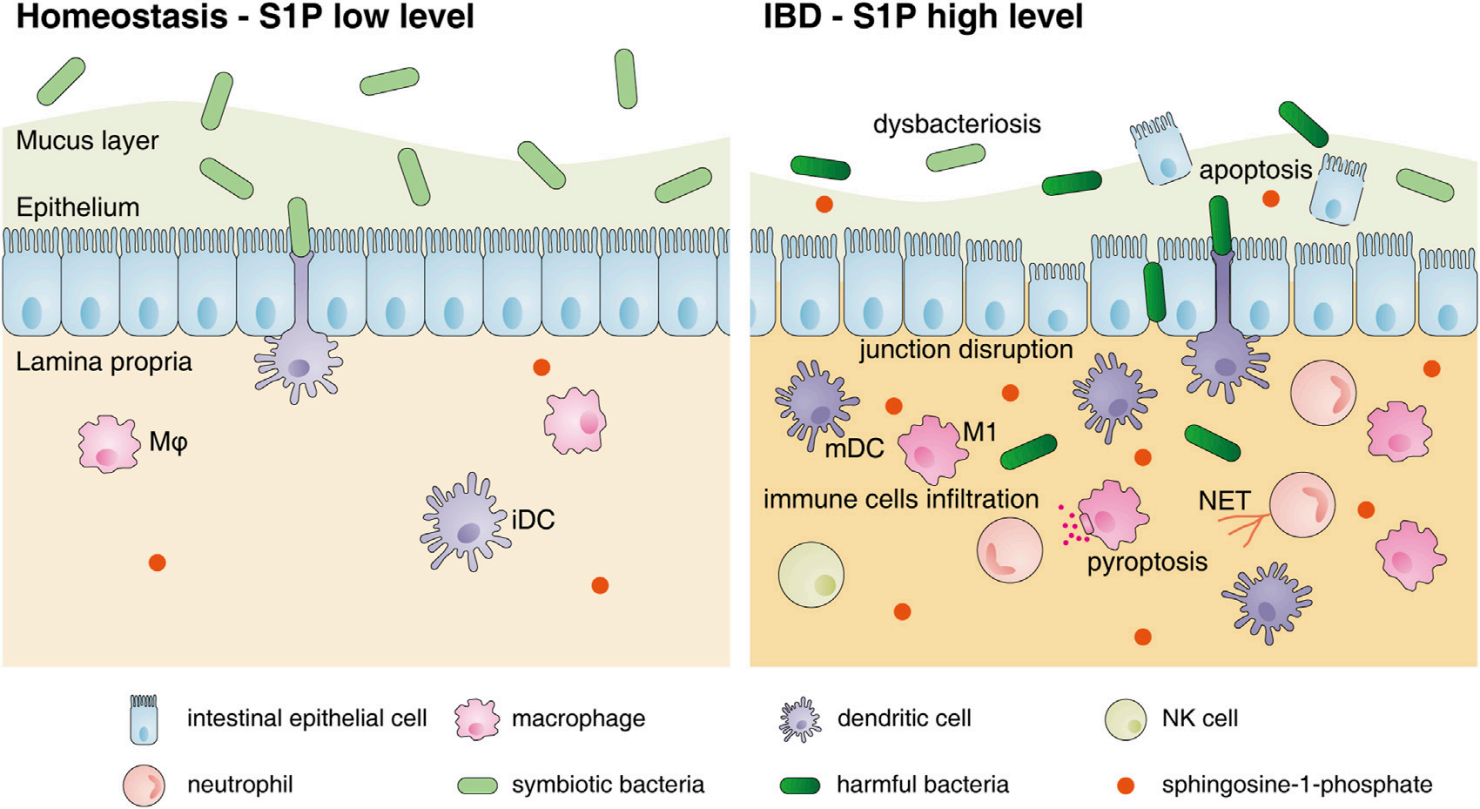
Changes in the intestinal mucosal microenvironment and S1P functions under IBD. The changes in the intestinal mucosal microenvironment under IBD conditions are as follows: decreased commensal bacterial diversity, increased IEC apoptosis, destroyed intercellular connections and increased infiltration of immune cells, including neutrophils, macrophages, DCs and NK cells, in the lamina propria. S1P can restore the connections between IECs and inhibit their apoptosis to protect the intestinal epithelial barrier. However, S1P also promotes the migration of various innate immune cells to inflamed sites, induces neutrophils to form NETs, stimulates M1 polarization and accelerates DC activation to aggravate intestinal inflammation. Abbreviations: S1P, sphingosine-1-phosphate; Mφ, macrophage; iDC, immature dendritic cell; mDC, mature dendritic cell; M1, classically activated macrophages; NET, neutrophil extracellular trap.

Based on the above studies, the regulatory effect of S1P-S1PR signaling on the intestinal mucosa is proinflammatory. This was further confirmed by performing animal experiments using PF-543 (a SphK1 inhibitor), which inhibits DSS-induced colitis (Liu and Jiang, 2020), and gut-specific knockout of S1PL, which leads to increased S1P levels in the colon and aggravates IBD symptoms (Suh and Saba, 2015). However, it should be noted that the use of 4-deoxypyridoxine hydrochloride and 2-acetyl-4 (tetrahydroxybutyl) imidazole, which markedly increase local intestinal S1P levels, attenuates CD-like chronic ileitis in TNF^△ARE^ mice by suppressing thymocyte maturation (Karuppuchamy et al., 2020). These different roles of S1P reveal the complicated functions of S1P in distinct biological processes. In addition, it should not be ignored that the S1P-S1PR axis can also promote the proliferation and repair of epithelial cells and may protect the intestinal epithelial barrier (Figure 2). Huang et al. demonstrated that epithelial-specific knockout of S1PP2 in mice led to milder colitis, lower levels of TNF-α, IL-6 and IL-1β, and higher mucosal integrity than that in WT mice after DSS induction, suggesting the protective effect of epithelial S1P on the intestinal mucosal barrier under acute colitis (Huang et al., 2016). However, the effects of S1P on the intestinal epithelial barrier under chronic colitis are still poorly understood.

S1PR modulators, represented by fingolimod and etrasimod or ozanimod, which more selectivity targets S1PR1, cause the internalization, degradation and subsequent downregulation of this receptor (Jo et al., 2005). These drugs have a profound anti-inflammatory effect by preventing the migration of lymphocytes into peripheral tissues. Ozanimod and etrasimod have been tested for effectiveness and safety in IBD patients in several clinical trials (Wang et al., 2022a). However, less attention has been focused on the direct effects of S1PR modulators on the intestinal epithelium. SEW2871, an S1PR1 agonist, has been proven to be therapeutic in IL-10 gene-deficient mice by reducing IEC apoptosis and improving epithelial barrier function (Dong et al., 2015). Additionally, S1PR1 was proven to inhibit TNF-α/cycloheximide-induced IEC apoptosis in a cell experiment (Greenspon et al., 2009), indicating the protective role of S1PR1 on the intestinal epithelium under inflammation. Thus, it is valuable to explore the effect of S1P modulators on IECs when S1PR1 signaling in IECs is inhibited. In addition, S1PR2, another S1P receptor that is independent of the pharmacology of S1PR modulators, is involved in various cell functions in the intestine. Chen et al. revealed the general pathogenic role of S1PR2 in a DSS-induced colitis mouse model (Chen et al., 2022). S1PR2 upregulates the expression of serum TNF-α and IL-18, as well as IL-18 in colon tissue, which is consistent with its proinflammatory effects on innate immune cells discussed above. However, as the most highly expressed S1PR in IECs under homeostasis, S1PR2 promotes the proliferation of IECs and protects the epithelial barrier. These contradictory results suggest the complicated regulatory functions of S1P in the intestinal microenvironment and that cell-specific inhibition of S1P may need to be considered in the development of new drugs to avoid inhibiting intestinal epithelial repair during anti-inflammation periods.

The crosslink between IECs and immune cells in the lamina propria is an important field in IBD research. The cytokines and factors secreted from epithelial cells or immune cells in the lamina propria can mutually achieve message transfer and thus impact signal transduction and cellular function. Our previous studies also confirmed that miR-590-3p in M2 macrophage-derived exosomes could promote epithelial cell regeneration and alleviate DSS-induced mucosal damage by activating the LATS1/YAP/β-Catenin pathway in IECs (Deng et al., 2021). S1P, as a bioactive molecule acting on cell membranes of its own or adjacent cells by autocrine or paracrine mechanisms to activate downstream pathways, may act as a “signal transmitter” between IECs and immune cells. Studies have confirmed that the phagocytosis activity of alveolar macrophages was positively correlated with the expression level of Spns2 in bronchial epithelial cells, suggesting the regulatory role of epithelial-derived Spns2 on macrophages (Tran et al., 2017). Exosomes can be produced by various types of cells and construct signal interactions between different cells. Enteropathogenic BSPs can stimulate SphK1 expression in MC38 cells and then increase the expression of S1P in IEC-derived exosomes (Deng et al., 2015). Moreover, the biological effects of S1P contained in intestinal mucosa-derived exosome-like nanoparticles promoted colon cancer in mice (Deng et al., 2015), suggesting the important regulation of S1P in the epithelium and lamina propria during gut inflammation and cancer.

## 4 S1Ps involvement in IBD treatment

S1PR modulators, as a novel kind of small molecule drug, have been considered potential therapies for IBD (Verstockt et al., 2022). The side effects of fingolimod, including heart rate reduction and temporary delay in atrioventricular conduction, due to its nontherapeutic binding with S1PRs in the cardiovascular system, limit its clinical use in IBD (Camm et al., 2014). However, this shortcoming was well overcome with the emergence of selective S1PR modulators targeting S1PR1, such as ozanimod and etrasimod, which have been used for IBD treatment. Ozanimod was approved by the US FDA and the European Commission for the treatment of UC in 2021, while further clinical trials are ongoing to confirm its efficacy and safety in adult and pediatric UC and CD patients. Based on previous studies, UC patients who received either ozanimod or etrasimod showed a higher incidence of clinical remission and response than those who received placebo (Sandborn et al., 2021; Vermeire et al., 2021; Sandborn et al., 2023). Ozanimod is also considered “a promising new player” due to its ideal curative effects in the treatment of CD (Feagan et al., 2020; Sleutjes et al., 2020). Adverse reactions to ozanimod and etrasimod that need attention include worsening of UC/CD, infection, and anemia and elevated liver aminotransferase levels (Feagan et al., 2020; Sandborn et al., 2021; Vermeire et al., 2021; Sandborn et al., 2023). Cardiovascular side effects were not significant and included transient, asymptomatic and low-grade atrioventricular block in only a few patients who received etrasimod (Sandborn et al., 2020). Altogether, S1PR modulators are promising to open a new era of IBD treatment due to their unique therapeutic targeting.

In addition to targeting S1PR1, increasing studies have also suggested the therapeutic effects of other factors in the S1P-S1PR pathway on IBD, but they were still limited to preclinical studies. PF543, a SphK1-specific inhibitor, improved colitis symptoms in a DSS-induced colitis mouse model and downregulated IL-1β and IL-6 mRNA levels in the colon (Sun et al., 2019b). Another SphK1-specific inhibitor, LCL351, has similar therapeutic effects on DSS-induced mice (Pulkoski-Gross et al., 2017). Knockout of the S1P transporter Spns2 significantly improved DSS- or oxazolone-induced colitis in mice (Donoviel et al., 2015). The S1PL inhibitor 4-deoxypyridoxine can reduce CD4^+^CD8^+^ double-positive immature thymocytes in the thymus and lead to the accumulation and apoptosis of mature thymocytes in the thymus medulla, thereby exerting a central immunosuppressive effect and improving CD-like ileitis in TNF^ΔARE^ mice (Karuppuchamy et al., 2020). In addition to receptor inhibitors targeting S1PR1, the S1PR2 inhibitor JTE-013 significantly reduced the histological score of colon inflammation in a DSS-induced colitis mouse model (Chen et al., 2022). Therefore, all factors in the SphK-S1P-S1PR pathway might serve as therapeutic targets for IBD, while because of the wide distribution of S1PRs in the body and the distinct effects mediated by S1PRs in cells, higher receptor selectivity should be considered in the development of S1P-related drugs.

Many IBD treatments have been found to be associated with S1P-S1PRs. Mesalazine reduced colon inflammation by decreasing the serum level of S1P in DSS-treated mice (Sun et al., 2019b). Stem cell therapy is a new treatment method for IBD (Misselwitz et al., 2020). Adipose-derived mesenchymal stem cell transplantation significantly downregulates SphK1 mRNA levels in the colonic mucosa of TNBS-induced mice to resolve colitis, and SphK1 mRNA can be further decreased by combination therapy with mesenchymal stromal cells and sulfasalazine (Yousefi-Ahmadipour et al., 2019). Metformin can reduce oxazolidone-induced colitis by downregulating plasma SphK1-S1P activity in rats (El-Mahdy et al., 2021). In addition, some active ingredients in plants, such as hesperidin and baicalin, have also been confirmed to alleviate DSS-induced colitis by inhibiting the SphK1-S1P-S1PR signaling pathway (Shafik et al., 2019; Yao et al., 2020).

## 5 Conclusion

In conclusion, S1P, a bioactive molecule, is widely distributed in the intestinal mucosa and its expression is increased in IBD. It exerts different biological effects on various cell types in the intestinal mucosa. Increased S1P can disturb the structural balance of the gut microflora and promote the development of colitis. For intestinal epithelial cells, S1P can promote cell proliferation and migration, inhibit cell apoptosis and strengthen intercellular connections to protect the intestinal epithelial barrier. For innate immune cells, S1P is prone to exert a proinflammatory effect to trigger and aggravate inflammation. S1P can promote the migration of immune cells to inflamed sites, inhibit the apoptosis of neutrophils, induce M1 macrophage polarization, and promote the maturation and activation of DCs to enhance the production of proinflammatory cytokines. SphK-S1P-S1PR pathway-associated drugs were newly used in clinical and animal studies for IBD treatment. In summary, beyond the role of S1P in lymphocyte trafficking, S1P exerts important and complicated effects on the regulation of different cell functions in the mucosal microenvironment of colitis. Selective S1P receptor modulators are needed to avoid inhibition of epithelial repair when exerting their anti-inflammatory functions in immune cells. Further in-depth and larger studies could focus on this interesting regulation of S1P in the intestinal mucosa and its roles in gut inflammation and IBD.

## Abbreviations

BSP, bacteria-secreted particle; CD, Crohn’s disease; Cer, ceramide; DC, dendritic cell; IBD, inflammatory bowel disease; IEC, intestinal epithelial cell; ISC, intestinal stem cell; M1, classically activated macrophage; M2, alternative activated macrophage; S1P, sphingosine-1-phosphate; S1PR, sphingosine-1-phosphate receptor; SM, sphingomyelin; Spns2, spinster 2; Sph, sphingosine; SphK1/2, sphingosine kinase 1/2; S1PL, sphingosine-1-phosphate lyase; S1PP1/2, sphingosine-1-phosphate phosphatase 1/2; UC, ulcerative colitis.
